# Multi-walled carbon nanotubes as reusable boosters of pyocyanin production for anticancer research

**DOI:** 10.1007/s00253-025-13543-w

**Published:** 2025-07-14

**Authors:** Joanna Honselmann genannt Humme, Kamila Dubrowska, Magdalena Perużyńska, Marek Droździk, Radosław Birger, Martyna Jurkiewicz, Tomasz Kędzierski, Ewa Mijowska, Tomasz Idzik, Jacek G. Sośnicki, Elżbieta Filipek, Mateusz Piz, Rafał Rakoczy, Adrian Augustyniak

**Affiliations:** 1 Department of Chemical and Process Engineering, Faculty of Chemical Technology and Engineering, West Pomeranian University of Technology in Szczecin, Szczecin, Poland; 2 Department of Experimental & Clinical Pharmacology, Pomeranian Medical University in Szczecin, Szczecin, Poland; 3 Department of Chemical Organic Technology and Polymeric Materials, Faculty of Chemical Technology and Engineering, West Pomeranian University of Technology in Szczecin, Szczecin, Poland; 4 Department of Nanomaterials Physicochemistry, Faculty of Chemical Technology and Engineering, West Pomeranian University of Technology in Szczecin, Szczecin, Poland; 5 Department of Organic and Physical Chemistry, Faculty of Chemical Technology and Engineering, West Pomeranian University of Technology in Szczecin, Szczecin, Poland; 6 Department of Inorganic and Analytical Chemistry, Faculty of Chemical Technology and Engineering, West Pomeranian University of Technology in Szczecin, Szczecin, Poland; 7 Center for Advanced Materials and Manufacturing Process Engineering (CAMMPE), Szczecin, Poland

**Keywords:** Carbon nanotubes, Melanoma, Phenazines, *Pseudomonas aeruginosa*, Stimulation

## Abstract

**Abstract:**

Carbon nanotubes (CNTs) emerged as nanomaterials with a wide variety of applications, e.g., as boosters of bioprocesses efficiency. The stimulation of the production of the blue pigment called pyocyanin is one of numerous examples. Moreover, its importance comes from the potential anticancer properties of the pigment. Therefore, this contribution evaluated different commercially available multi-walled carbon nanotubes (MWCNTs) in pyocyanin production using the Design of Experiment methodology. The interactions between pigment-producing bacteria and nanomaterials were revealed as well. Moreover, the purified pigment was tested against normal and cancer cell lines. Interestingly, the results showed that all tested CNTs stimulated pyocyanin production. The most effective CNTs were used in the process optimisation in terms of temperature (32 °C) and carbon nanomaterial concentration (812 μg/mL). It was also revealed that the optical density and viability of the bacterial culture were elevated, while the pyoverdine production was decreased. Furthermore, no oxidative stress was detected. Moreover, the confocal microscopy study indicated that the cells surrounded the aggregates of MWCNT and produced more proteins within the biofilm structure, compared to the control experiment. The tests on neoplastic cell lines showed an excellent antiproliferative activity of pyocyanin against melanoma without pronounced adverse effects on normal fibroblasts. The nanomaterial incorporated in the bioprocess was successfully reused, making the method sustainable and cost-effective.

**Key points:**

• *The stimulative effect of nanomaterial on pyocyanin production was optimised*

• *Nanomaterial can be reused in the bioprocess without losing the stimulative effect*

• *Pyocyanin exhibits significant antiproliferative action against melanoma*

**Graphical abstract:**

**Figure:**
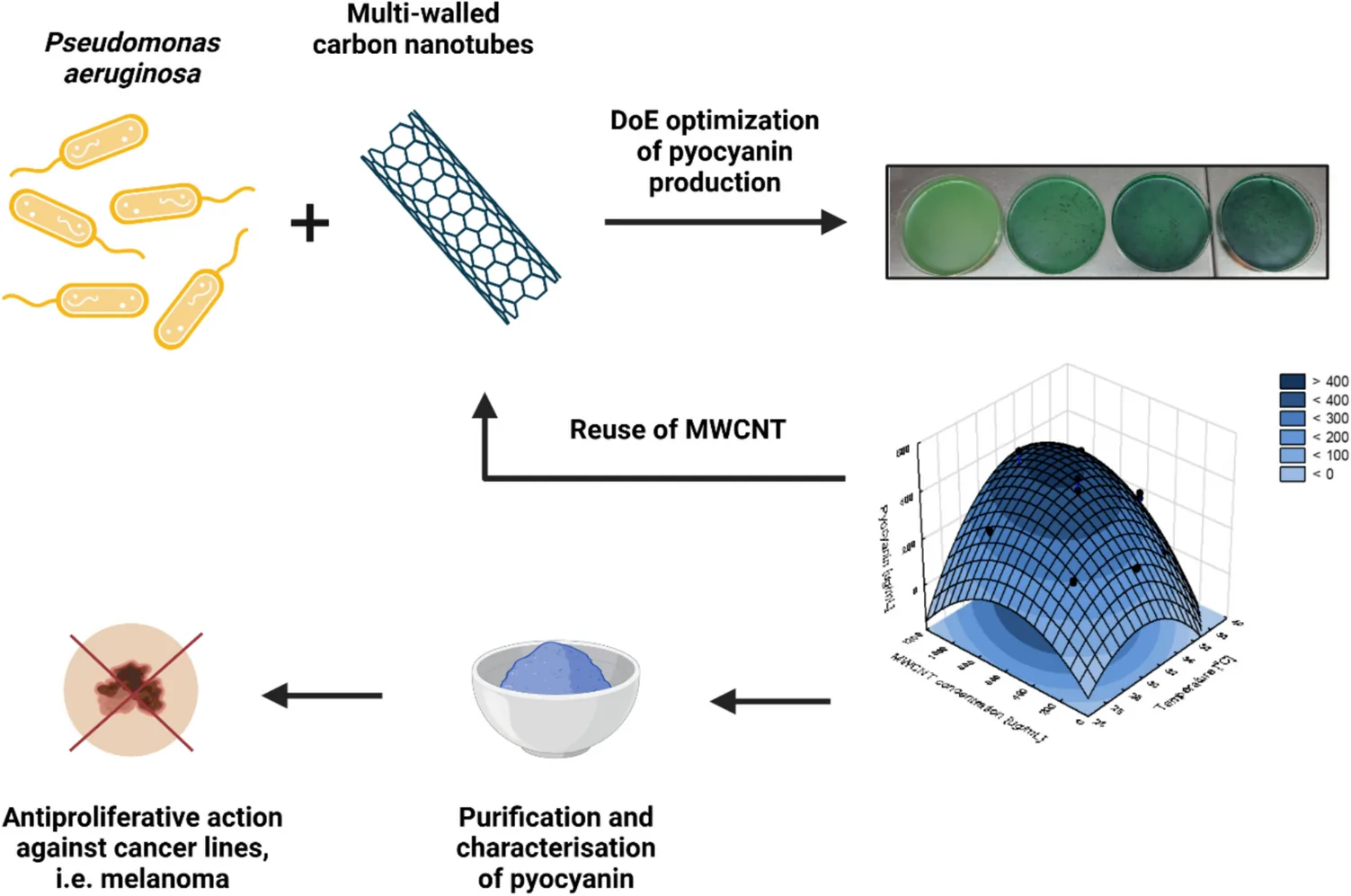

**Supplementary Information:**

The online version contains supplementary material available at 10.1007/s00253-025-13543-w.

## Introduction

Carbon nanotubes (CNTs) are widely used carbon nanomaterials (Rathinavel et al. 2021). Due to their excellent electrical and thermal conductivity and mechanical properties, they have found numerous applications, e.g. in batteries and capacitors (Jun et al. 2023), in polymer composites (as a reinforcement) (Licht et al. 2024) or in antifouling paints (Dustebek et al. 2016). However, new biotechnological applications are also emerging. Among them, CNTs can be utilised in the adsorption of organic pollutants (Mishra and Sundaram 2023; Mishra et al. 2023), increased generation of bioelectricity (Jourdin et al. 2014; Antonucci et al. 2022), fabrication of bio-based composites (Valentini et al. 2016), increasing methane production (Li et al. 2015), construction of biosensors (Kumar et al. 2023) or in biological imaging and drug delivery (Hernández-Rivera et al. 2016; Sonowal and Gautam 2024). Recently, it has been shown that MWCNTs may have a stimulative effect on pyocyanin (PYO) production (Jabłońska et al. 2022), a blue pigment produced by *Pseudomonas aeruginosa*. In the last decade, the interest in pyocyanin has grown due to its antimicrobial and anticancer properties, potential agricultural application as a biocontrol agent or improvement of microbial fuel cells by mediating electron transfer (Jabłońska et al. 2023). Especially anticancer properties are of a growing interest and have been, so far, investigated on breast cancer (Patil et al. 2017; Abdelaziz et al. 2022; Al-Jassani et al. 2023; Shouman et al. 2023; Marey et al. 2024) (CAL-15 and MCF-7, MD-MB-231 cell lines), colorectal cancer (Koyun et al. 2022; Shouman et al. 2023; Marey et al. 2024) (HT-29, Colo2, HCT-116), liver cancer (Zhao et al. 2014; Patil et al. 2017; Shouman et al. 2023) (HepG2), lung cancer (Patil et al. 2017; Kohatsu et al. 2020; Marey et al. 2024) (A549), leukaemia (Kohatsu et al. 2020) (HL-60), pancreatic cancer (Moayedi et al. 2018) (Panc-1), cervical cancer (Patil et al. 2017; Shouman et al. 2023) (HeLa), prostate cancer (Shouman et al. 2023) (PC3), and melanoma (Patil et al. 2017; Koyun et al. 2022) (SK-MEL-2 and SK-MEL-30) cell lines. For these reasons, intensification of pyocyanin and more effective production are desired. However, to date, no standardised production methods for pyocyanin are available, which hinders its efficient production and further commercial use. Another question is whether MWCNTs could be reused in the subsequent bioprocesses.

In this work, we aimed to compare the stimulative effects of MWCNTs from different commercially available sources to reveal the best available option. The application of selected nanotubes in pyocyanin production has been further optimised using statistical planning of the experiment. Under optimised conditions, we also assessed the physiological state of bacteria (*Pseudomonas aeruginosa* ATCC 27853) under exposure to nanomaterial, purified the pigment and evaluated its anticancer response with the use of following cancer cell lines: human malignant melanoma cells (A375), human lung adenocarcinoma cells (A549), human hepatoma cells (HepG2) and human breast adenocarcinoma cells (MCF7). Lastly, we explored the possibility of reusing the MWCNT in multiple production cycles.

## Materials and methods

Multi-walled carbon nanotubes used in this research are commercially available and originated from different producers (Sigma Aldrich — SA, CheapTubes — ChT, Nanocyl — NC). The nanomaterials were analysed using various analytical methods. Raman spectroscopy analysis was performed to determine the vibronic properties of the chemical bonds in nanomaterials (InVia Renishaw, UK; excitation wavelength of 532 nm). The phase composition was determined by X-ray diffraction (XRD) using a Cu Kα radiation source (Aeris, Malvern Panalytical, Malvern, UK). The Brunauer–Emmett–Teller (BET) method calculated the specific surface area and pore size distribution. N_2_ adsorption/desorption isotherms were acquired at liquid nitrogen temperature (77 K) using a Micromeritics ASAP 2460 (Norcross, GA, USA). Scanning electron microscopy (SEM) with an energy dispersive spectroscopy (EDS) detector (Apreo 2S, Thermo Fisher Scientific, USA) with a beam excitation voltage of 20 kV was utilised to determine the chemical composition of nanomaterials. Transmission electron microscopy (TEM) analysis was performed (Spectra 300, Thermo Fisher Scientific, USA) with a beam excitation voltage of 300 kV to assess the morphology of the MWCNTs. The ash content was determined using thermogravimetric analysis (TGA, TG 209 F1 Libra).

All MWCNTs were suspended in distilled water, heated to 100 °C for 30 min, and sonicated for another 30 min before application. The bacterial strain used in this research was *Pseudomonas aeruginosa* ATCC 27853. All experiments were led in King’s A broth (10 mL) in stationary conditions.

### The influence of different MWCNTs on pyocyanin production

The preliminary investigation of the influence of MWCNT from different producers was conducted in 96-well plates (total volume of 100 µL, 37 °C, 48 h) to assess how their concentration affected the optical density (OD) and viability of the cells. The cultures were also visually examined due to their pronounced green-blueish colour. After that, the most promising results (i.e. 500 and 1000 µg/mL of MWCNT addition) were researched in the bigger volume (10 mL, 37 °C, 48 h) on the Petri dish. Among the measurements were the OD, viability and pyocyanin production.

### The optimisation of the pyocyanin production process

To optimise the production process of pyocyanin, the Design of Experiment (DoE) approach was employed with MWCNT concentration and process temperature as the independent factors. The chosen plan was based on the Central Composite Plan. Each experiment was conducted in three replicates. The range of the tested MWCNT concentration covered 175.74 to 1024.26 µg/mL, and the temperature range was from 24.93 to 39.07 °C. After 72 h of culturing, the samples were centrifuged and subjected to chloroform-hydrochloric acid extraction. The concentration of pyocyanin was calculated from the calibration curve. The created plan of experiments is presented in Table 1.

**Table 1:** Plan of experiments created with DoE

Run	2**(2) central composite, *nc* = 4 ns = 4 n0 = 2 runs = 10	
	Temperature (°C)	MWCNT concentration (μg/mL)
1	27.00	300.00
4	37.00	900.00
10 (C*)	32.00	600.00
3	37.00	300.00
2	27.00	900.00
6	39.07	600.00
9 (C*)	32.00	600.00
7	32.00	175.74
8	32.00	1024.26
5	24.93	600.00

C* — centre point

### Pyocyanin adsorption on MWCNT

To investigate the adsorption of pyocyanin on MWCNT, the control sample (King’s A medium with 100 µg/mL of dissolved pyocyanin powder) and the tested sample (King’s A medium with 100 µg/mL of dissolved pyocyanin powder and 812 µg/mL of suspended MWCNT) were incubated at 32 °C for 24 h. After that, the samples were centrifuged (10,000 rpm, 10 min) to separate the supernatant from the sediment containing MWCNT. The absorbance of the supernatants and the pure medium with pelleted MWCNT was quantified through pyocyanin extraction using the chloroform-hydrochloric acid method.

### Basic physiology of the optimised culture

Basic measurements of the culture parameters were performed to investigate the physiological state of bacteria contacted with an optimised concentration of MWCNTs. Optical density (OD), viability and pyoverdine (fluorescent siderophore) production were assessed. Shortly, the OD was measured spectrophotometrically at a wavelength of 600 nm. Viability was estimated using a resazurin reduction assay (also interpreted as metabolic activity indication (Králová et al. 2024), 520/590 nm), and pyoverdine was monitored fluorometrically directly in the culture (398/460 nm). All methods were applied at the time points of 2, 4, 6, 8, 10, 12, 24, 48 and 72 h in three biological and three technical replicates, with samples carefully withdrawn to avoid the aspiration of the nanomaterials.

Additional parameters to be investigated were using the primary carbon source (glycerol), dry biomass production and pyocyanin production assayed after 24, 48 and 72 h. The results were used to calculate the process yields at different time points.

ROS generation was investigated using the DCFH-DA assay. Shortly, the optimised and the control cultures were incubated for 8 h (before pyocyanin production started). Later, the cultures were centrifuged, washed with PBS and resuspended with DCFH-DA reagent (100 µM) for 30 min. After that time, the cells were centrifuged and rewashed with PBS. The OD was unified for both types of cultures, and the measurements were conducted at 485/530 nm.

The activity of superoxide dismutase (SOD) was monitored using the SOD Colorimetric Activity Kit (EIASODC, Thermo Fisher Scientific, USA), according to the previously described method (Honselmann genannt Humme et al*.*, 2024).

### CLSM analyses of the bacteria-nanomaterial interactions

The cultures were examined with confocal laser scanning microscopy (CLSM), Leica Stellaris 5 (Leica, Wetzlar, Germany), to assess the viability and arrangement of the cells. It was performed by adding 100 µL of the culture to the 18-well plate and incubating it for 16 h at 32 °C. Later, the supernatant was removed from the wells, and two types of staining were performed. LIVE/DEAD analysis was performed by rinsing the biofilm with PBS and staining it with a LIVE/DEAD kit for 15 min, PBS rinsing again and covering the samples with AntiFade. Matrix staining was performed using SYPRO™ Ruby (proteins), concanavalin + Alexa 633 (exopolysaccharides) and POPO-3 (cells and eDNA) dyes. The biofilm was rinsed with deionised water, stained simultaneously with all dyes for 30 min, rinsed with water again and covered with a glycerol-based antifade agent. The three-channel analysis was performed simultaneously under × 630 magnification. In all samples, the laser energy was set equally for each stain. Image analysis and visualisation were performed on LasX software (Leica).

### NMR and FT-IR characterisation of pyocyanin

To obtain the pyocyanin as a pure powder, it was extracted from the culture with chloroform, then re-extracted with 0.2 M HCl, neutralised with NaOH and again re-extracted with a small volume of chloroform. The samples were mixed with anhydrous sodium sulphate (VI) to remove the water residues and dried in the vacuum drier. The obtained powders were subjected to NMR and FTIR-ATR analyses to confirm their identity and purity.

^1^H, ^13^C NMR spectroscopic measurements were performed on a Bruker DPX 400 Avance III HD spectrometer, operating at 400.2 and 100.6 MHz, respectively. TMS was used as an internal standard, and spectra were acquired in 5 mm probes at 21 °C. The standard Bruker pulse programs were used in the case of ^1^H and ^13^C NMR. For ^1^H NMR measurements, the most important parameters were number of scans: 16, relaxation delay: 1 s and spectral width: 8012.8 Hz. In the case of ^13^C NMR measurements, the most important measurement parameters were the number of scans: 16,384, relaxation delay: 2 s and spectral width: 24,038.5 Hz. The MestReNova (version: 12.0.4) program was used for NMR analyses. For detailed peak assignments, 2D spectra were acquired using standard Bruker software (^1^H, ^1^H NOESY, ^1^H, ^1^H DFQCOSY, ^13^C and 1H COSY). The Lorentz-to-Gauss resolution enhancement was applied to refine coupling constants since the routine ^1^H NMR spectra did not allow direct determination. For this transformation, MestReNova (version: 12.0.4) was used by ticking both exponential and Gaussian multiplication functions and introducing a negative value for the exponential line broadening (− 3.5 Hz) and a positive value for the Gaussian parameter (1.3 Hz). In the description of NMR spectral data, the standard abbreviation for multiplicities was used (s = singlet, d = doublet, dd = doublet of doublets and ddd = doublet of doublets of doublets).

FTIR-ATR analysis of samples obtained in pyocyanin powder was performed in the wavelength range of 400–4000 cm^−1^ using a Nicolet iS5 spectrophotometer (ThermoFisher, USA). The measurements were conducted at a resolution of 4 cm^−1^, and the number of scans was 32. OMNIC SPECTRA software was used to perform the analysis and data collection.

### Anticancer properties of pyocyanin

#### Antiproliferative study

The antiproliferative activity of PYO was assessed using the Cell Proliferation Reagent (WST-1 assay). In this study, normal murine fibroblast cells (L929), human malignant melanoma cells (A375), human lung adenocarcinoma cells (A549), human hepatoma cells (HepG2) and human breast adenocarcinoma cells (MCF7) were seeded in 96-well plate at density depending on the doubling time of individual cell lines and duration of the experiment. L929, A375 and A549 cells were cultured in DMEM high glucose (Sigma-Aldrich Merck Group, USA) supplemented with 10% heat-inactivated fetal bovine serum (FBS, EURx, Poland), 2 mM L-glutamine (Corning, USA) and penicillin–streptomycin (Corning, USA). In contrast, HepG2 and MCF7 were cultured in MEM (Corning, USA) supplemented as above and additionally with nonessential amino acids (1 ×) (Corning, USA). After 24 h, the culture medium was removed and replaced with fresh medium containing PYO at final concentrations equal to 1, 10, 25, 50, 75, 100, 150 and 200 µM for 24 and 72 h. Due to its high sensitivity, only in the case of the A375 cell line was it necessary to modify the tested concentrations: 0.1, 1, 10, 25, 50, 75, 100 and 150 µM. Initially, PYO was dissolved in DMSO (Sigma-Aldrich Merck Group, USA) at 100 mM, aliquoted and frozen (final concentrations of DMSO in the medium did not exceed 0.2%; the initial impact of 0.2% DMSO on cell viability was negatively verified). The cells without tested compounds in the culture medium (but with DMSO) were used as a control, and the tested compounds in the medium without cells were used as blank. After 24/72 h, WST-1 reagent was added and incubated with the cells for 30 min, and absorbance was measured at 450 nm (with 620 nm background correction) using a spectrophotometric microplate reader (Infinite 200 Pro, Tecan, Switzerland). The cell viability was calculated using the following formula: [(*A*_test_ − *A*_blank_)/(*A*_control_ − *A*_blank_)] × 100%. The readings were acquired from at least four independent experiments (each conducted in triplicate). The IC50 values (the inhibitory concentration causing 50% growth inhibition) were evaluated using an online calculator (AAT Bioquest, Inc., Quest Graph™ IC50 Calculator (v.1) retrieved from: https://www.aatbio.com/tools/ic50-calculator (accessed on: 26.07.2024).

#### The cytotoxicity study

After 24 h of treatment, CytoTox96 Non-Radioactive Cytotoxicity Assay (Promega, USA) was used to assess lactate dehydrogenase (LDH) leakage under the above-mentioned conditions. The release of intracellular LDH into the culture medium indicates irreversible cell death due to cell membrane damage. Aliquots of 50 µL from all the test and control wells were transferred after treatment into fresh 96-well flat clear bottom plates, following the manufacturer’s protocol. The absorbance was measured at 490 nm using a spectrophotometric microplate reader (Infinite 200 Pro, Tecan, Switzerland). Untreated cells were used as the negative control, while cells treated for 45 min with Lysis Solution (0.9% Triton X-100) were used as the positive control (maximum LDH release) — the tested compounds in a medium without cells served as blanks. Readings were acquired from three independent experiments (each conducted in triplicate). The percentage viability was calculated using the following formula: viability [%] = 100 − (experimental LDH release – blank)/(maximum LDH release – blank) × 100%. Optical microscopy imaging of L929, A375, A549, HepG2 and MCF7 cells after 24 h of treatment was performed using a Smart Fluorescent Cell Analyzer Microscope JuLi (Korea).

### Reuse of MWCNTs

The reuse of the MWCNTs incorporated in the cultures in the subsequent processes of pyocyanin production was attempted. Firstly, the optimised and the control cultures were conducted in 300 mL in 300 cm^2^ T-flasks with filtered caps. After 72 h of incubation, the cultures were centrifuged, and pyocyanin was extracted and quantified according to the above-mentioned method. The centrifuged biomass with MWCNTs was dried for 24 h at 100 °C to inactivate the cells and calcinated at 500 °C to remove the biomass. The obtained material was ground with a mortar and pestle to obtain uniform consistency and used in another batch of pyocyanin production. Such an approach was conducted in three rounds (i.e. pure MWCNTs, MWCNTs after the first use and MWCNTs after the second use). The nanomaterials were analysed employing Raman spectroscopy, XRD, BET, SEM–EDS, TEM and TGA analyses (as mentioned above).

## Results and discussion

### The influence of different MWCNTs on pyocyanin production

The comparison of the influence of different MWCNTs on the OD in microplate cultures showed significant stimulation in the case of all tested MWCNTs in comparison to the control (Fig. S1, from 125 to 1000 µg/mL for NC, from 250 to 1000 µg/mL for SA and 500 µg/mL for ChT). Moreover, the observed colour of the cultures corroborated the hypothesis that high concentrations of MWCNTs stimulated pyocyanin production. However, the viability values were not entirely in line with the OD measurements, which might be caused by a reduction in the assay’s sensitivity caused by a high concentration of black MWCNTs in wells (Augustyniak et al. 2022). The significant stimulation of the viability was noted concerning the control in the case of 500 µg/mL for NC, 250 µg/mL for SA and 1000 µg/mL for ChT. On the other hand, lower signals (but not significantly different from the control) were noted for 1000 µg/mL for NC and 500 and 1000 µg/mL for SA. It could be accounted for by fluorescence obstruction in a 96-well plate caused by MWCNTs.

**Fig. 1:**
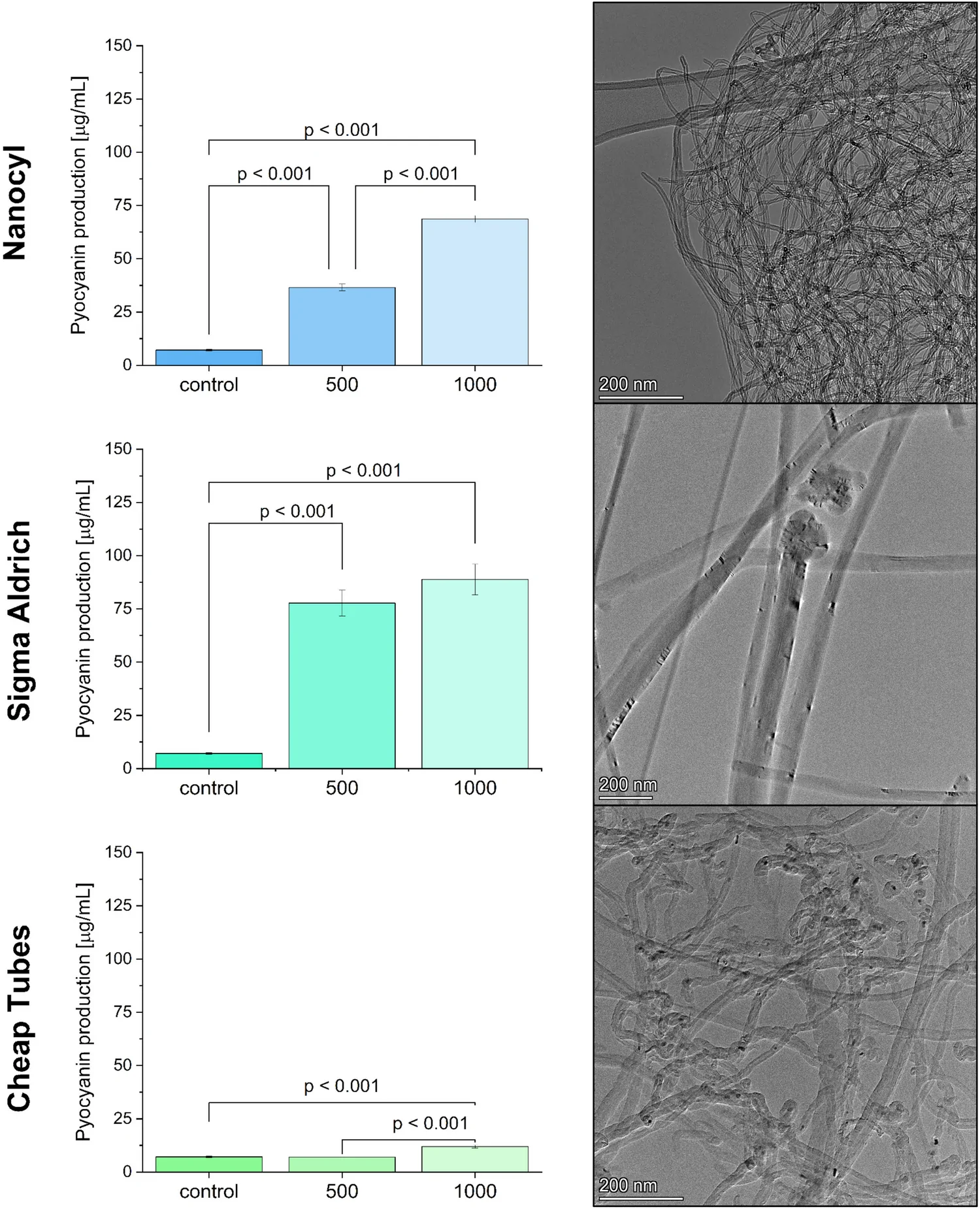
The influence of different commercial MWCNTs on pyocyanin production of *P. aeruginosa* in Petri dish cultures (37 °C, 48 h) (left panels) and their corresponding TEM images (right panels)

Based on the most promising results (expressing the highest stimulation), the same assays were performed in the cultures led in the Petri dish in a volume of 10 mL. The selected concentrations of MWCNTs for these experiments were 1000 and 500 µg/mL. The OD (measured in a way that reduces the risk of aspiration of CNT) was significantly increased in the samples with NC 1000 µg/mL, compared to the control (Fig. S2). Interestingly, other types of MWCNT did not cause significant differences from the control. Similar results were obtained in the case of viability. It was significantly higher in the cultures with NC 1000 µg/mL, compared to the control. On the other hand, pyocyanin production (Fig. 1) was significantly stimulated in the case of NC 1000 and 500 µg/mL, SA 1000 and 500 µg/mL and ChT 1000 µg/mL, which indicated that the level of culture viability in the resazurin assay was not proportional to pyocyanin production. The most pronounced stimulation was noted for 1000 µg/mL of SA, reaching 88.79 µg/mL, compared to the control, producing only 7.12 µg/mL of pyocyanin. Based on these results, the pyocyanin production process was optimised using SA MWCNTs, as they had shown the highest potential to stimulate pyocyanin production effectively. TEM images presented in Fig. 1 (right panels) show that SA MWCNT morphology differs from NC MWCNTS and ChT MWCNTs. It is composed of a very straight and rigid structure with tiny inner space and an outer mean diameter of ~ 90 nm. Their diameter is also the most developed, compared to other samples. NC MWCNTs and ChT MWCNTs are typical spaghetti-like structures with outer mean diameters of ~ 15 and ~ 25 nm, respectively.

### Optimisation of pyocyanin production using MWCNTs as process stimulators

**Fig. 2:**
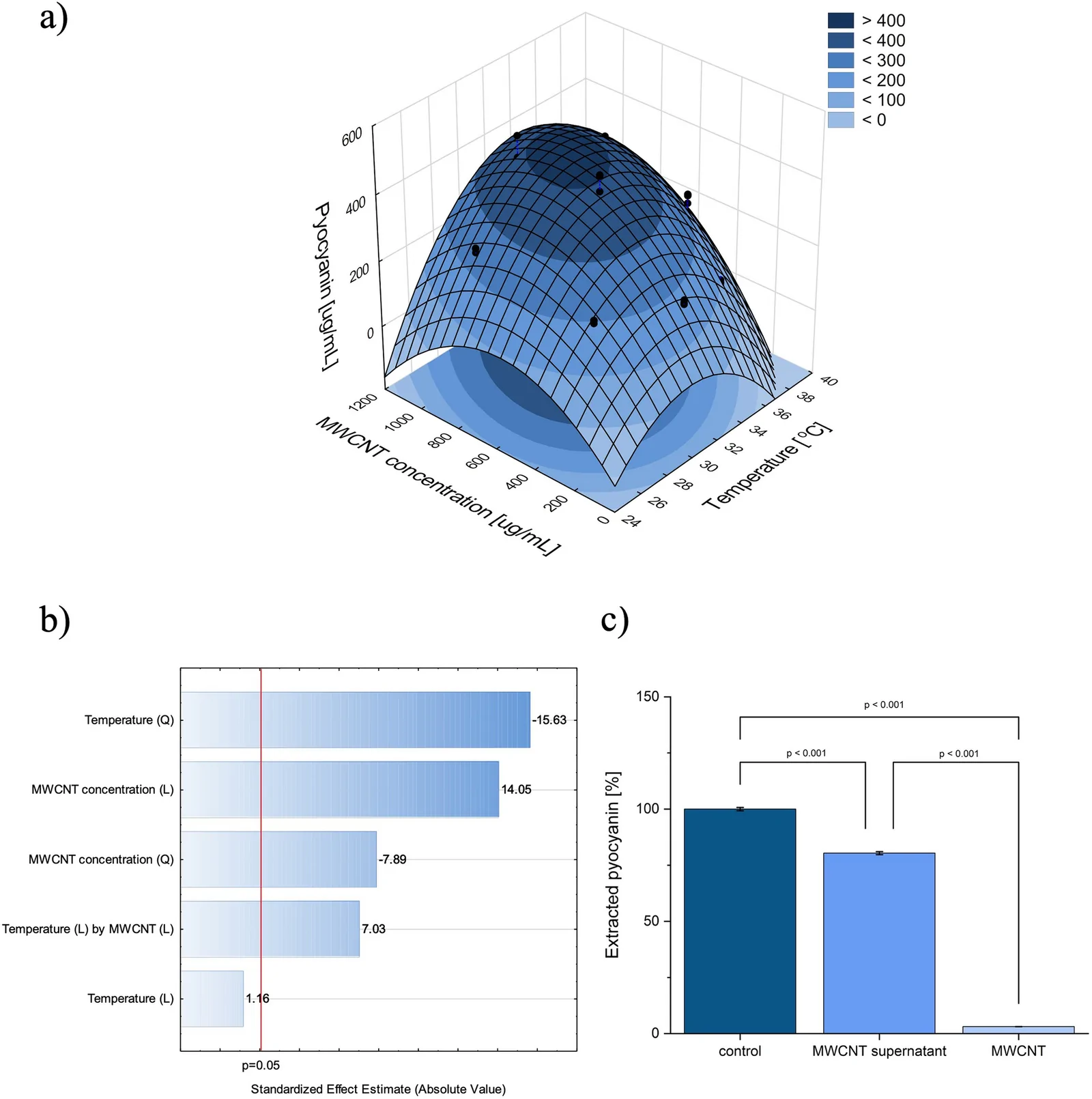
Pyocyanin production **a** DoE optimisation; **b** Pareto chart; and **c** pyocyanin adsorption on MWCNT

The experiments conducted according to the DoE plan enabled the fitting of the function and assessing which factors significantly influenced pyocyanin production (Fig. 2a). The obtained fit was high and equalled *R*^2^ adj. = 0.944. Among the factors significant for pyocyanin production were the temperature (although only quadratic), MWCNT concentration (linear and quadratic) and the interaction between the two factors (Fig. 2b). The optimal MWCNT concentration and process temperature were calculated, and they equalled to 812.13 µg/mL and 32 °C with predicted pyocyanin concentration of 421.02 μg/mL. The obtained temperature agreed with our previous optimisations of pyocyanin production (Honselmann genannt Humme et al*.* 2024).

### Pyocyanin adsorption on MWCNT

The concentration of pyocyanin extracted after incubation with MWCNTs (without bacteria) was lower in comparison to the control (around 80% of the control) (Fig. 2c). Further separation attempts performed on the MWCNT pellet released only 3% of the control concentration. Based on these findings, it was concluded that pyocyanin is partially adsorbed on MWCNT. However, the fact that complete extraction of pyocyanin from the MWCNT pellet was impossible may indicate chemical MWCNT-pyocyanin binding. Pyocyanin may induce the creation of H_2_O_2_, e.g. by oxidating NADPH in cells (Abdelaziz et al. 2023). Interestingly, ROS may increase the sorption capabilities of MWCNTs, as was shown on fulvic acids, by treating this carbon nanomaterial with H_2_O_2_ (Czech 2017). The adsorption of pyocyanin on various materials was previously reported, e.g. on clays (including montmorillonite, sepiolite and palygorskite) or carbon materials, which additionally confirms this possibility (Fashina and Deng 2022; Hirakawa et al. 2022). Based on the obtained results, it can be hypothesised that partial adsorption of pyocyanin on MWCNT may be one of the reasons for elevated pyocyanin production. Pyocyanin production is associated with quorum sensing (QS) in *P. aeruginosa*, regulated primarily by autoinducers such as Acyl-homoserine lactone (AHL) and *Pseudomonas* quinolone signal (PQS). Therefore, there is a possibility that the adsorption of pyocyanin and/or these signals could also overregulate production (Abdelaziz et al. 2023). PQS signals affect the operation of LasR-LasI and RhlR-RhlI systems, which contribute to increasing levels of *Pseudomonas* autoinducers (PAI-1 and PAI-2), positively affecting *phz* operons (Mudaliar and Bharath Prasad 2024). On the other hand, reducing the quantity of another AHL (3-oxo-C12-HSL) could remove the inhibitory factor of phenazine synthesis (Sun et al. 2016). Lastly, the sorption of pyocyanin could reduce the negative signal in a feedback loop, forcing cells to overproduce the pigment. Since phenazine synthesis pathways in *P. aeruginosa* are tightly associated with QS, more than one mechanism may be involved in the observed outcome (Mudaliar and Bharath Prasad 2024). Nevertheless, the mechanisms underlying the stimulation of PYO production need to be verified in further research, preferably in transcriptomic analyses, which were outside our study’s goal.

### Basic characterisation of the optimised culture

The optimised and control cultures were compared based on biomass/substrate, product/biomass and product/substrate yields at different time points (24, 48 and 72 h) (Fig. S3a). Differences were noted for biomass/substrate yield at 24 and 48 h; it was higher for the control culture than the optimised culture and 72 h higher for the optimised culture than for the control. The more pronounced differences were noted in the case of product/biomass and product/substrate yields. Product/biomass yield reached 35.44 µg of PYO/mg of biomass and 117.02 µg of PYO/mg of biomass for the control and optimised culture after 72 h, respectively (around 3.3 times higher for the optimised culture). Product/substrate yield reached 7.08 µg of PYO/mg of glycerol and 20.41 µg of PYO/mg of glycerol for the control and optimised culture after 72 h, respectively (around 2.9 times higher for the optimised culture).

**Fig. 3:**
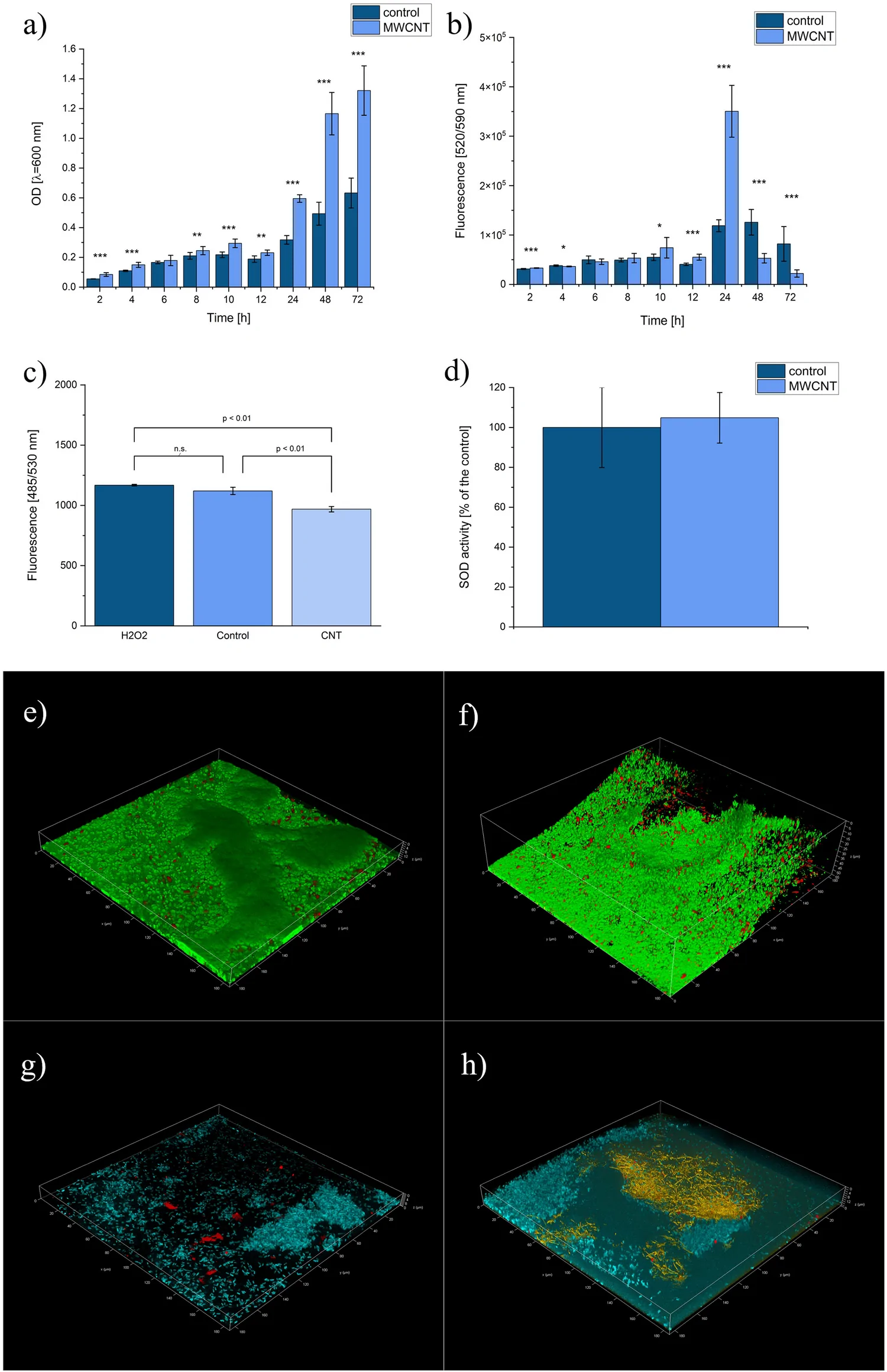
The characterisation of the optimised and control culture: **a** OD, **b** viability, **c** ROS, **d** SOD, **e** CLSM LIVE/DEAD staining of the control, **f** LIVE/DEAD staining of the MWCNT-exposed culture (green — live cells; red — dead cells), **g** matrix staining of the control, and **h** matrix staining of the MWCNT-exposed culture (blue — eDNA and cells, red — exopolysaccharides, and yellow — proteins)

The monitoring of the OD (Fig. 3a) revealed a lasting trend of increased OD values in the case of MWCNT-supplemented culture, compared to the control. However, the viability of the cells was changing (Fig. 3b). The stimulative effect of MWCNT on the viability of cells was noted at the 2nd, 10th, 12th and 24th h of the incubation. The viability was higher in the control culture at the 4th, 48th and 72nd h of the incubation. Such results indicate that the cells incubated with MWCNT reached their viability peak after 24 h and proceeded with the death phase much faster than the control culture. It was previously reported that adding MWCNTs to *Geobacter* cocultures resulted in an accelerated metabolic rate (Zheng et al. 2020). The pyoverdine monitoring (Fig. S3b) revealed increased fluorescence values in the control culture (in comparison to the MWCNT-supplemented sample) at the 4th, 6th, 8th, 24th, 48th and 72nd h. It may suggest that the culture incubated with MWCNT is reprogrammed to produce pyocyanin instead of pyoverdine. Our previous research noted similar findings where pyocyanin production was stimulated with zinc oxide nanoparticles, resulting in lower pyoverdine-associated fluorescence (Honselmann genannt Humme et al*.* 2024).

The DCFH-DA assay revealed lowered ROS levels in the case of MWCNT-exposed culture when compared to the control and H_2_O_2_-spiked culture (Fig. 3c). This may suggest that the nanomaterial presence did not elevate the ROS and reduced their naturally occurring presence. These findings were confirmed in the SOD assay, where no significant differences in the enzyme activity were found between the control and MWCNT culture (Fig. 3d). The possibility of interference between reagents and MWCNT was excluded in these assays due to the sample collection that avoided the aspiration of nanomaterial agglomerates (see “Basic physiology of the optimised culture”).

### CLSM analyses of the bacteria-nanomaterial interactions

The interactions between microorganisms, especially bacteria, and CNTs have been extensively studied with frequent reporting of antibacterial properties of CNTs, especially single-walled carbon nanotubes (SWCNTs) (Selim et al. 2024). On the other hand, multi-walled carbon nanotubes (MWCNT) were less frequently reported as bactericidal, and some studies have even underlined different effects associated with bacteria-MWCNT interactions. Among them are the aggregation effect of *Pseudomonas aeruginosa* cells contacted with MWCNT (Kovach et al. 2020) and enhanced conjugative plasmid transfer (Weise et al. 2022). In our research, the CLSM analysis of the biofilm revealed the differences between the control and MWCNT-exposed cultures (Fig. 3e–h). LIVE/DEAD staining allowed observing the cells surrounding the aggregates of carbon nanotubes and an apparent increase in the thickness of the biofilm layer (the aggregates are not visible due to a lack of staining; their presence was confirmed in the white field optical mode). Interestingly, no pronounced changes in cell viability were noticed between the control and MWCNT-treated samples (Fig. 3e, f). In the case of the biofilm, matrix staining showed that in the presence of MWCNTs, more pronounced production of proteins took place, especially under the nanomaterial aggregate (Fig. 3g, h). It was suggested that exopolysaccharide-binding proteins (CdrA) can play a role in biofilm formation in *P. aeruginosa*, where, in the initial stage, they act as matrix cross-linkers (Courtney et al. 2020).

### NMR and FT-IR analyses

**Fig. 4:**
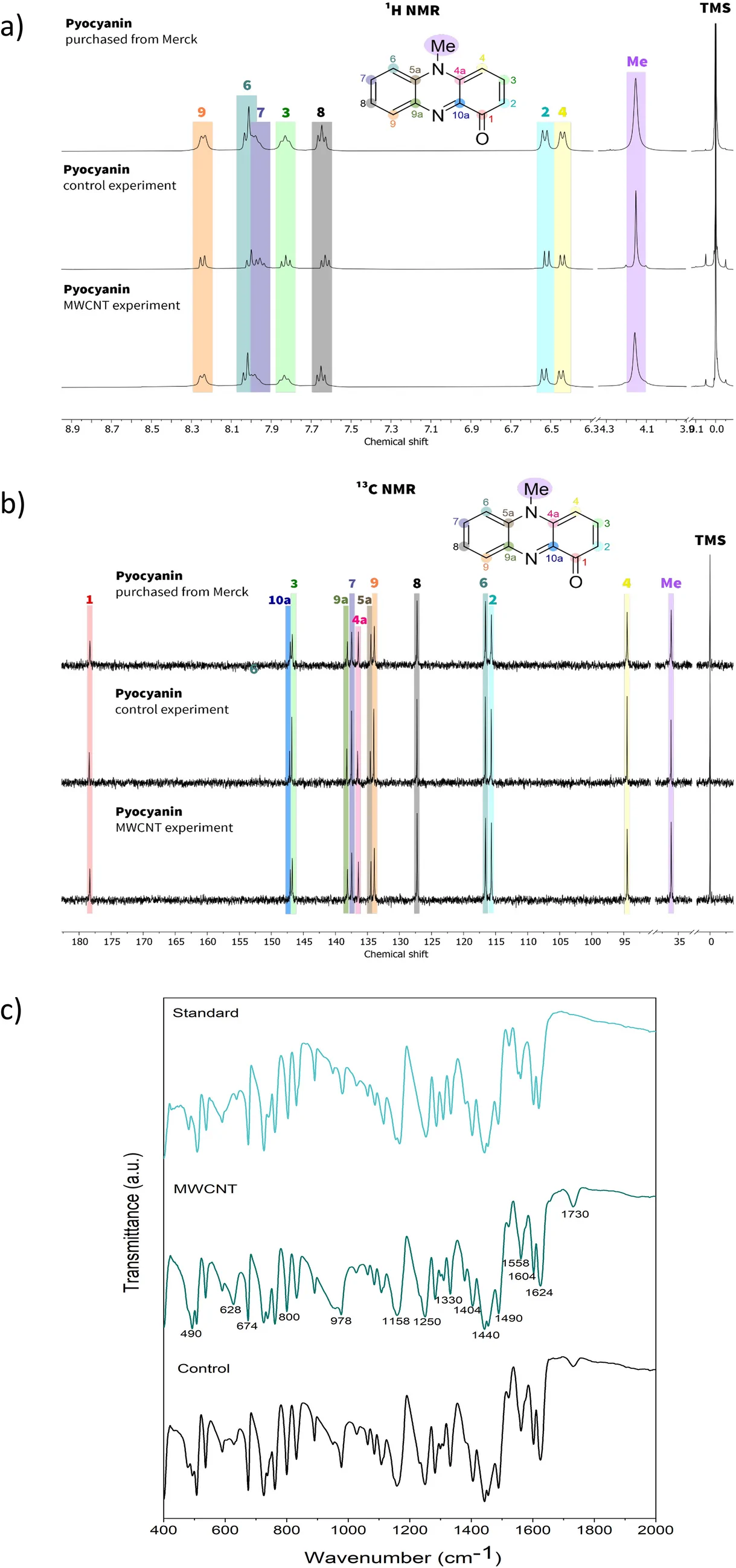
Analysis of the purified pyocyanin: **a** summary of ^1^H NMR, **b** summary of ^13^C NMR, **c** FTIR-ATR analyses of the purified pyocyanin (400–2000 cm.^−1^)

The samples obtained from both the control experiment and the one utilising MWCNTs were analysed using ^1^H and ^13^C NMR methods. The resulting spectra were further compared with those of a sample sourced commercially. Notably, all spectra recorded for 2 mg of pyocyanin dissolved in 0.5 ml of perdeuterated methanol (CD_3_OD) exhibited remarkable similarity, indicating structural consistency. However, our assignments of the signals to the corresponding atoms, based on the Overhouser effect, differ from those reported in the literature. Figure 4 a and b graphically present a summary of the ^1^H NMR and ^13^C NMR spectra of the three pyocyanin samples with the assignment of the resonance signals to the corresponding atoms based on our measurements and literature data. Description of NMR data are as follows: ^1^H NMR (400 MHz, MeOD) δ 4.16 (s, 3H, N-CH_3_), 6.45 (d, *J* = 8.2 Hz, 1H, H-4), 6.53 (d, *J* = 8.9 Hz, 1H, H-2), 7.65 (dd, *J* = 8.3, 8.3 Hz, 1H, H-8), 7.83 (dd, *J* = 8.2, 8.9 Hz, 1H, H-3), 7.98 (dd, *J* = 8.3, 8.6 Hz, 1H, H-7), 8.03 (d, *J* = 8.6 Hz, 1H, H-6), 8.25 (d, *J* = 8.3 Hz, 1H, H-9).^13^C {H} NMR (101 MHz, CD_3_OD) *δ* 36.11 (N-CH_3_), 94.46 (C-4), 115.64 (C-2), 116.57 (C-6), 127.25 (C-8), 133.93 (C-9), 134.46 (C-5a), 136.42 (C-4a), 137.45 (C-7), 138.12 (C-9a), 146.73 (C-3), 147.02 (C-10a) and 178.32 (C-1). Additional fragments of spectra are available in Supplementary materials (Fig. S4–S6).

FTIR-ATR spectra of pyocyanin powder samples obtained from the control (Control) and test (MWCNT) samples were compared with IR spectra of commercial pyocyanin labelled (Standard). From the IR spectra shown, it can be seen that qualitatively, they are almost identical in the entire range of wavelength numbers, i.e. 400–4000 cm^−1^ (Supplementary materials, Fig. S7). Minor differences relate only to the intensity of the recorded absorption bands. Figure 4 c shows a fragment of the compiled IR spectra in the 400–2000 cm^−1^ range because absorption bands with the highest intensity and with well-developed extremes characteristic of, among other things, stretching bond vibrations in standard pyocyanin are recorded in this range. From numerous literature data (Mahmoud et al. 2016; DeBritto et al. 2020; Hamad et al. 2020), it is known that the bands registered in the 2000–1500 cm^−1^ wavenumber range (with extrema at ~ 1730, 1624, 1604 and 1558 cm^−1^) are mainly related to stretching vibrations, of various types of double bonds in pyocyanin, i.e. C = O, C = C and C = N, and those registered below 1500 cm^−1^ (with extrema registered, among others, at ~ 1490, 1440, 1404, 1330, 1250, 1158 and 978 cm^−1^) are bands of stretching vibrations of single bonds, e.g. C–C, C–N and C–H. Many bands in the 1500–400 cm^−1^ range also correspond to deformation vibrations. Bands associated with skeletal stretching vibrations of C = C bonds are located in the 1620–1450 cm^−1^ range, and bands of deformation vibrations of C–H bonds occur below 900 cm^−1^ (Hamad et al. 2020). The band not recorded in the IR spectrum of standard pyocyanin with an extremum at ~ 1730 cm^−1^, according to the literature, is associated with stretching vibrations of C = C bonds in the aromatic ring of pyocyanin (Mahmoud et al. 2016). Based on the analysis of the IR spectra of the tested samples, it can be undoubtedly concluded that the test sample (MWCNT) contains pure pyocyanin with a well-developed structure.

### Anticancer properties

**Fig. 5:**
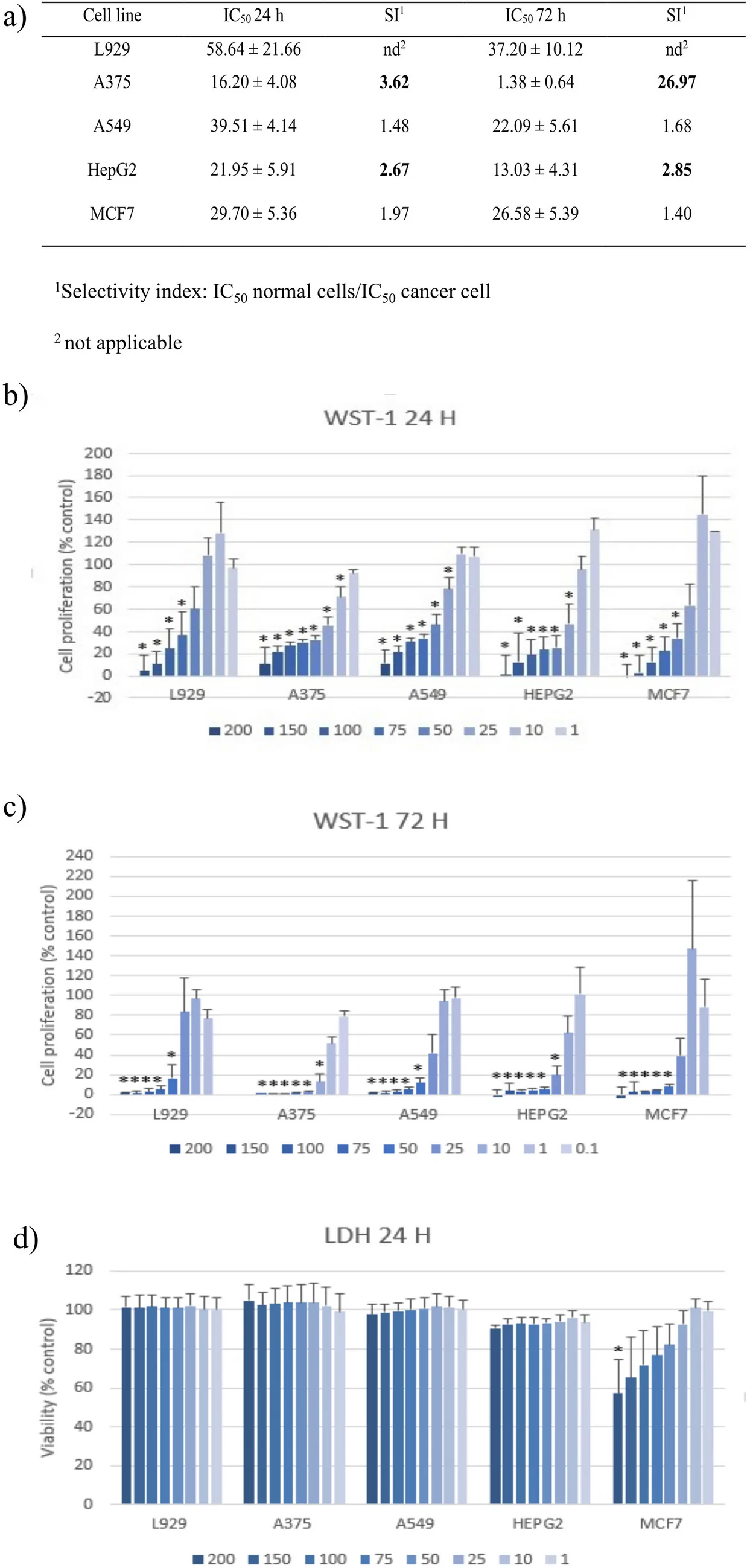
Anticancer assays: **a** the IC_50_ values (µM) and selectivity-index (*SI*) determined using WST-1 assay after 24 and 72 h of PYO treatment; **b** the antiproliferative activity of PYO determined using WST-1 assay after 24 h and **c** 72 h of treatment; **d** the cytotoxic activity of PYO determined using LDH assay after 24 h of treatment. The results are expressed as the *mean* and *SD* from at least four (b, c) or three (d) independent experiments; * *p* < 0.05 vs. control (Student’s *t*-test)

The Cell Proliferation Reagent (WST-1 assay) was used to evaluate the antiproliferative activity of PYO against selected human neoplastic cell lines, including melanoma (A375), lung cancer (A549), hepatoma (HepG2), breast cancer (MCF7) and murine fibroblasts (L929) as non-cancerous cells. The results are summarised as IC50 values in Fig. 5 a and cell proliferation (% control) in Fig. 5 b and c. According to the IC50 values, the cell sensitivity was as follows: A375 > HepG2 > MCF7 > A549 > L929 (after 24 h of treatment). A similar relationship (HepG2 > MCF7 > HCT > A549) was demonstrated by Ibrahim et al. after 48 h of incubation with PYO (Khaled Ibrahim et al. 2024). In the current study, small differences (higher sensitivity of HepG2 cells) were observed after 72 h, as the sensitivity of cell lines to PYO could be ranked as follows: A375 > HepG2 > A549 > MCF7 > L929. Undoubtedly, the most sensitive cell line defined was melanoma. Its sensitivity to pyocyanin was also confirmed on other melanoma cell lines (SK-MEL-30 and SK-MEL-2) (Patil et al. 2017; Koyun et al. 2022). The selectivity index (SI) also confirmed that melanoma cells were unique (after 72 h) to meet the PYO selectivity criteria (*SI* > 10) proposed by Peña-Morán (Peña-Morán et al. 2016). However, based on the selectivity criteria proposed by Kohatsu et al. (Kohatsu et al. 2020) (*SI* > 2), the anticancer activity of PYO against HepG2 cells can also be considered selective.

The LDH test results did not confirm the above observations (Fig. 5d). This test was performed only after 24 h of PYO treatment because the test is dedicated to assessing acute toxicity, which is measured by the amount of LDH released into the culture medium only from cells with damaged cell membranes. The stability of the measured LDH (up to 9 h) limits the feasibility of the test to short-time incubations with tested compounds. In the tested concentration range, it was impossible to determine IC50 values based on the LDH test results. A significant reduction in viability was observed only in MCF7 cells treated with 200 µM of PYO. These seemingly inconsistent results can be explained by differences in the functional mode of both tests, which were confirmed by microscopic observation (Figure S8, Supplementary materials). In the case of all cell lines, a decrease in culture density was observed in comparison to the control (at a concentration-dependent cell sensitivity, namely for A375 from a concentration of 10 µM). This microscopic observation was confirmed by the WST-1 assay results, which allowed us to determine the changes in the number of viable, metabolically active cells in the culture. However, only in the case of MCF7 (starting from a concentration of 50 µM) characteristic features of dead cells were observed in the culture (predominance of spherical cells). The results indicate that PYO has antiproliferative rather than cytotoxic effects on cancer cells, but a more detailed understanding of the mechanism of action of PYO requires further research, which exceeds the current goal of the study.

### Reuse of MWCNT

**Fig. 6:**
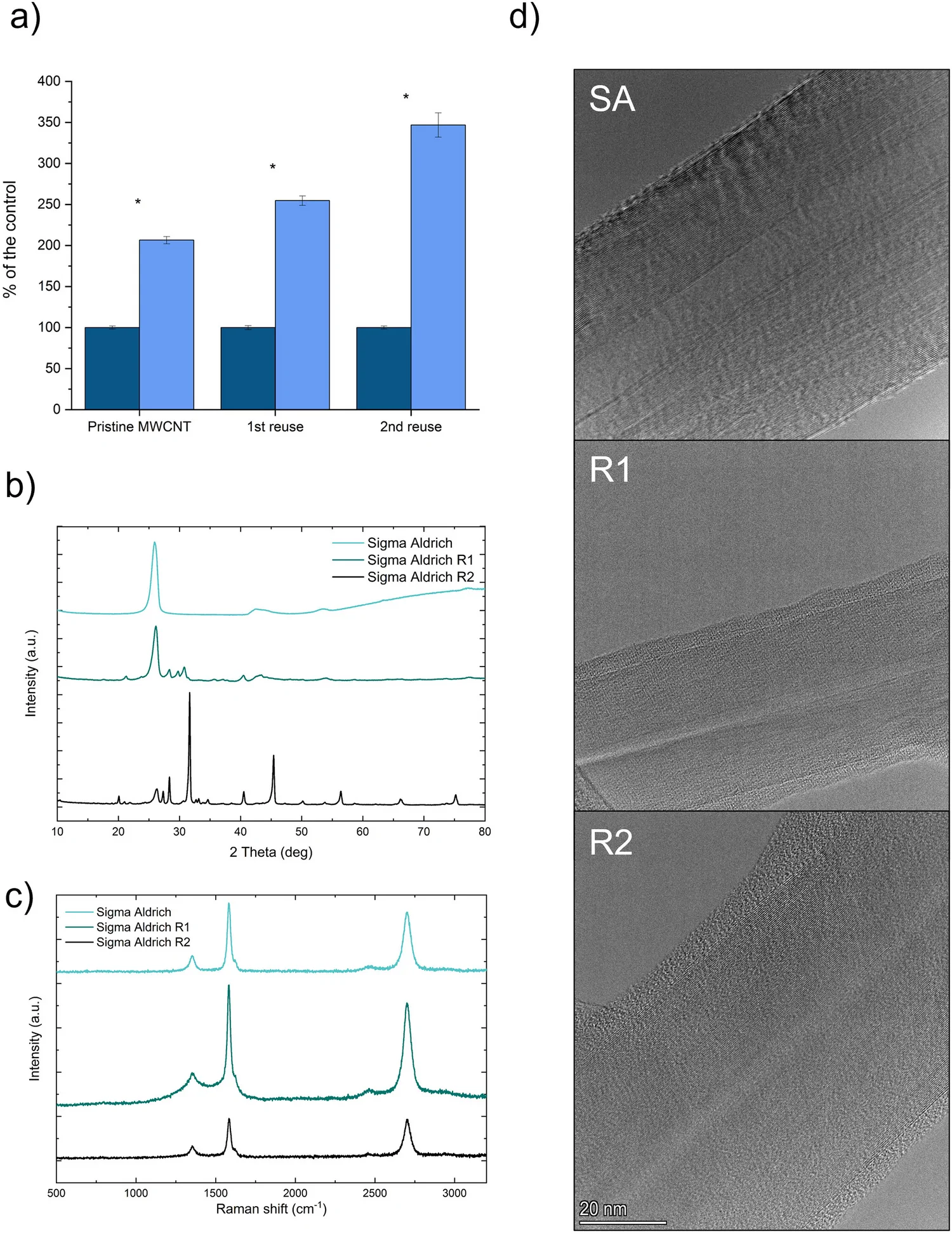
The reuse of the MWCNTs: **a** production of pyocyanin with reused MWCNTs,** b** XRD analysis of the reused MWCNTs, **c** Raman spectroscopy, **d** TEM micrographs (SA — pristine MWCNTs, R1 — MWCNTs reused once, R2 — MWCNTs reused twice)

An attempt was made to reuse the MWCNT incorporated in the pyocyanin production process to reduce its cost and decrease the possible negative environmental impact. It was demonstrated that the nanomaterial could be reused at least two times without losing the stimulative effect (even obtaining higher stimulation) on pyocyanin production (Fig. 6a). Interestingly, the nanotubes did not require any specific regeneration to maintain their bioactivity in the following processes.

The analyses of the reused Sigma Aldrich MWCNTs showed a decrease in the specific surface area (25.74, 16.62 and 4.5 m^2^/g, respectively) and total pore volume (0.01087, 0.00795 and 0.00072 cm^3^/g, respectively) with each use cycle. Nevertheless, the increasing content of ashes (7.05, 25.94 and 32.64%, respectively) was noted, and the higher the ash content, the higher the stimulation of pyocyanin production. SEM–EDX analysis (Supplementary materials, Fig. S9) enabled the detection of only C in pristine Sigma Aldrich MWCNTs, C, O, P, S, Cl, K and Mg in MWCNTs that were reused once, and C, O, P, S, Cl, K, Mg and Fe in the sample reused twice. The biogenic elements O, P and S are probably leftover from the bacterial biomass, while the source of K, Fe and Mg is likely King’s A medium.

XRD analysis of the pristine MWCNTs identified three reflexes at 2*θ* = 26° (002), 43° (101) and 53° (004) (Fig. 6b). These reflexes are typical for the hexagonal graphite structure (Nawar et al. 2020). XRD analyses of the reused Sigma Aldrich nanomaterial showed a reflex typical for MWCNTs (26°) in each sample. However, the other reflexes (43° and 53°) were less pronounced with each reuse. Interestingly, after the second reuse of nanomaterial, some sharp reflexes were identified by the XRD software as inorganic salts, i.e. KCl and K_2_SO_4_.

Raman spectroscopy of the pristine nanomaterials identified three prominent bands: D band, G band and 2D band, which are characteristic of MWCNTs (Dobrzańska-Danikiewicz et al. 2017) (Fig. 6c). Raman spectroscopy of the reused MWCNTs revealed no pronounced differences between the pristine sample, reused for the first time and the one reused twice. For pristine MWCNTs the characteristic bands were identified at 1353 cm^−1^, 1582 cm^−1^ and 2699 cm^−1^, for the MWCNTs reused once at 1352, 1584 and 2700 cm^−1^ and for MWCNTs reused twice at 1353 cm^−1^, 1583 cm^−1^ and 2699 cm^−1^, respectively. The calculated *I*_D_/*I*_G_ ratios were 0.26, 0.32 and 0.34, respectively. It shows a slight increase in structural defects and/or contaminants in the samples after each application.

TEM analysis (Fig. 6d) showed no pronounced morphological differences between the pristine and reused MWCNTs. However, the images indicate the deposition of an amorphous layer on the outer surface of the nanotubes, which is thicker after each application in the pigment production process.

## Conclusions

We showed that MWCNTs can be applied as pyocyanin production boosters and that statistical tools can optimise this effect. The incubation of bacteria with MWCNTs resulted in differences in the optical density and viability of bacteria, which may suggest that the presence of nanomaterial positively influences the growth and metabolism of *P. aeruginosa*. Moreover, the aggregation of cells on MWCNTs was noted, and increased protein production in the vicinity of the nanomaterial was visualised. Moreover, no signs of oxidative stress were detected.

We also confirmed previously reported anticancer activity of the produced pyocyanin. Furthermore, our study confirmed the high selectivity of this pigment against melanoma cells. It was also confirmed that the action of pyocyanin is rather antiproliferative than cytotoxic. This insight may contribute to the further use of pyocyanin in cancer research.

The used nanomaterial could be recycled, making it more sustainable and cost-effective. Among the differences in the reused MWCNTs were the drop in specific surface area, increased ash content and changes in elemental composition.

In summary, an efficient and cost-effective method for the pyocyanin production process using MWCNTs was developed and proposed.

## Supplementary Information

Below is the link to the electronic supplementary material.

ESM1 (DOCX 8.20 MB)

## Data Availability

The data generated in this research is available in the Bridge of Knowledge data repository: 10.34808/d19q-x536.

## References

[CR1] Abdelaziz AA, Kamer AMA, Al-Monofy KB, Al-Madboly LA (2022) A purified and lyophilized *Pseudomonas aeruginosa* derived pyocyanin induces promising apoptotic and necrotic activities against MCF-7 human breast adenocarcinoma. Microb Cell Fact 21:262. 10.1186/s12934-022-01988-x 36528623 10.1186/s12934-022-01988-x PMC9759863

[CR2] Abdelaziz AA, Kamer AMA, Al-Monofy KB, Al-Madboly LA (2023) *Pseudomonas aeruginosa*’s greenish-blue pigment pyocyanin: its production and biological activities. Microb Cell Fact 22:110. 10.1186/s12934-023-02122-1 37291560 10.1186/s12934-023-02122-1 PMC10251607

[CR3] Al-Jassani MJ, Hizam MM, Shakir MN, Sadeq SS, Majeed MA, Alwan NH, Hassan ZF (2023) Onkologia I Radioterapia © Evaluation of the efficiency of pyocyanin purified from *Pseudomonas aeruginosa* as anticancer agent toward human breast cancer cell line CAL-51

[CR4] Antonucci A, Reggente M, Roullier C, Gillen AJ, Schuergers N, Zubkovs V, Lambert BP, Mouhib M, Carata E, Dini L, Boghossian AA (2022) Carbon nanotube uptake in cyanobacteria for near-infrared imaging and enhanced bioelectricity generation in living photovoltaics. Nat Nanotechnol 17:1111–1119. 10.1038/s41565-022-01198-x 36097045 10.1038/s41565-022-01198-x

[CR5] Augustyniak A, Dubrowska K, Jabłońska J, Cendrowski K, Wróbel RJ, Piz M, Filipek E, Rakoczy R (2022) Basic physiology of *Pseudomonas aeruginosa* contacted with carbon nanocomposites. Appl Nanosci 12:1917–1927. 10.1007/s13204-022-02460-3

[CR6] Courtney R, M JH, Michael M, Cynthis W, J WD, R PM (2020) The versatile *Pseudomonas aeruginosa* biofilm matrix protein CdrA promotes aggregation through different extracellular exopolysaccharide interactions. J Bacteriol 202. 10.1128/jb.00216-20 10.1128/JB.00216-20 PMC7484184 32661078

[CR7] Czech B (2017) The effect of MWCNT treatment by H2O2 and/or UV on fulvic acids sorption. Environ Res 155:1–6. 10.1016/j.envres.2017.01.037 10.1016/j.envres.2017.01.037 28167266

[CR8] DeBritto S, Gajbar TD, Satapute P, Sundaram L, Lakshmikantha RY, Jogaiah S, Ito S ichi (2020) Isolation and characterization of nutrient dependent pyocyanin from *Pseudomonas aeruginosa* and its dye and agrochemical properties. Sci Rep 10. 10.1038/s41598-020-58335-6 10.1038/s41598-020-58335-6 PMC6994680 32005900

[CR9] Dobrzańska-Danikiewicz AD, Wolany W, Łukowiec D, Jurkiewicz K, Niedziałkowski P (2017) Characteristics of multi-walled carbon nanotubes-rhenium nanocomposites with varied rhenium mass fractions. Nanomaterials and Nanotechnology 7:1847980417707173. 10.1177/1847980417707173

[CR10] Dustebek J, Kandemir-Cavas C, Nitodas SF, Cavas L (2016) Effects of carbon nanotubes on the mechanical strength of self-polishing antifouling paints. Prog Org Coat 98:18–27. 10.1016/j.porgcoat.2016.04.020

[CR11] Fashina B, Deng Y (2022) Smectite, sepiolite, and palygorskite for inactivation of pyocyanin, a biotoxin produced by drug-resistant *Pseudomonas aeruginosa*. Microporous and Mesoporous Materials 331. 10.1016/j.micromeso.2021.111668

[CR12] Hamad MNF, Marrez DA, El-Sherbieny SMR (2020) Toxicity evaluation and antimicrobial activity of purified pyocyanin from *Pseudomonas aeruginosa*. Biointerface Res Appl Chem 10:6974–6990. 10.33263/BRIAC106.69746990

[CR13] Hernández-Rivera M, Zaibaq NG, Wilson LJ (2016) Toward carbon nanotube-based imaging agents for the clinic. Biomaterials 101:229–240. 10.1016/j.biomaterials.2016.05.045 27294540 10.1016/j.biomaterials.2016.05.045

[CR14] Hirakawa H, Kimura A, Takita A, Chihara S, Tanimoto K, Tomita H (2022) Adsorption of extracellular proteases and pyocyanin produced by *Pseudomonas aeruginosa* using a macroporous magnesium oxide-templated carbon decreases cytotoxicity. Curr Res Microb Sci 3. 10.1016/j.crmicr.2022.100160 10.1016/j.crmicr.2022.100160 PMC9743004 36518171

[CR15] Honselmann genannt Humme J, Dubrowska K, Grygorcewicz B, Gliźniewicz M, Paszkiewicz O, Głowacka A, Musik D, Story G, Rakoczy R, Augustyniak A (2024) Optimised stress – intensification of pyocyanin production with zinc oxide nanoparticles. Microb Cell Fact 23:215. 10.1186/s12934-024-02486-y 39061071 10.1186/s12934-024-02486-y PMC11282796

[CR16] Jabłońska J, Dubrowska K, Augustyniak A, Wróbel RJ, Piz M, Cendrowski K, Rakoczy R (2022) The influence of nanomaterials on pyocyanin production by *Pseudomonas aeruginosa*. Appl Nanosci 12:1929–1940. 10.1007/s13204-022-02461-2

[CR17] Jabłońska J, Augustyniak A, Dubrowska K, Rakoczy R (2023) The two faces of pyocyanin - why and how to steer its production? World J Microbiol Biotechnol 39:103. 10.1007/s11274-023-03548-w 36864230 10.1007/s11274-023-03548-w PMC9981528

[CR18] Jourdin L, Freguia S, Donose BC, Chen J, Wallace GG, Keller J, Flexer V (2014) A novel carbon nanotube modified scaffold as an efficient biocathode material for improved microbial electrosynthesis. J Mater Chem A Mater 2:13093–13102. 10.1039/c4ta03101f

[CR19] Jun JH, Paeng J, Kim J, Shin J, Choi IS, Lee JH (2023) Intertwined CNT assemblies as an all-around current collector for volume-efficient lithium-ion hybrid capacitors. ACS Appl Mater Interfaces 15:25484–25494. 10.1021/acsami.3c02492 37199724 10.1021/acsami.3c02492

[CR20] Khaled Ibrahim M, Ahmed El-Zawhry Y, Abdel Rahman Esmaiel A, Abdel Rahman Askora A, Tohamy Mostafa MA (2024) Exploring the antimicrobial and anticancer potential of pyocyanin produced by *Pseudomonas aeruginosa* strain ONO14782. 10.21203/rs.3.rs-3996369/v1

[CR21] Kohatsu H, Kamo S, Furuta M, Tomoshige S, Kuramochi K (2020) Synthesis and cytotoxic evaluation of N-Alkyl-2-halophenazin-1-ones. ACS Omega 5:27667–27674. 10.1021/acsomega.0c04253 33134730 10.1021/acsomega.0c04253 PMC7594318

[CR22] Kovach K, Sabaraya IV, Patel P, Kirisits MJ, Saleh NB, Gordon VD (2020) Suspended multi-walled, acid-functionalized carbon nanotubes promote aggregation of the opportunistic pathogen *Pseudomonas aeruginosa*. PLoS One 15. 10.1371/journal.pone.0236599 10.1371/journal.pone.0236599 PMC7386566 32722685

[CR23] Koyun MT, Sirin S, Erdem SA, Aslim B (2022) Pyocyanin Isolated from *Pseudomonas aeruginosa*: characterization, biological activity and its role in cancer and neurodegenerative diseases. Brazilian Archives of Biology and Technology 65. 10.1590/1678-4324-2022210651

[CR24] Králová M, Patakyová S, Veselá M, Baudys M, Viktorová J, Krýsa J, Veselý M, Dzik P (2024) Resazurin assay as a suitable method for testing the antimicrobial activity of photocatalytic surfaces. J Photochem Photobiol A Chem 455:115769. 10.1016/j.jphotochem.2024.115769

[CR25] Kumar S, Sidhu HK, Paul AK, Bhardwaj N, Thakur NS, Deep A (2023) Bioengineered multi-walled carbon nanotube (MWCNT) based biosensors and applications thereof. Sensors and Diagnostics 2:1390–1413

[CR26] Li L-L, Tong Z-H, Fang C-Y, Chu J, Yu H-Q (2015) Response of anaerobic granular sludge to single-wall carbon nanotube exposure. Water Res 70:1–8. 10.1016/j.watres.2014.11.042 25499894 10.1016/j.watres.2014.11.042

[CR27] Licht G, Hofstetter K, Licht S (2024) Polymer composites with carbon nanotubes made from CO2. RSC Sustainability 2:2496–2504. 10.1039/d4su00234b

[CR28] Mahmoud S, Sayed E, Ziedan H, Farrag ES, Khalaphallah R (2016) Antifungal activity of pyocyanin produced by *Pseudomonas aeruginosa* against *Fusarium oxysporum* Schlech a root-rot phytopathogenic fungi

[CR29] Marey MA, Abozahra R, El-Nikhely NA, Kamal MF, Abdelhamid SM, El-Kholy MA (2024) Transforming microbial pigment into therapeutic revelation: extraction and characterization of pyocyanin from *Pseudomonas aeruginosa* and its therapeutic potential as an antibacterial and anticancer agent. Microb Cell Fact 23. 10.1186/s12934-024-02438-6 10.1186/s12934-024-02438-6 PMC11170807 38867319

[CR30] Moayedi A, Nowroozi J, Akhavan Sepahy A (2018) Cytotoxic effect of pyocyanin on human pancreatic cancer cell line (Panc-1). Iran J Basic Med Sci 21:794–799. 10.22038/ijbms.2018.27865.6799 10.22038/IJBMS.2018.27865.6799 PMC6118082 30186565

[CR31] Mishra S, Sundaram B (2023) Efficacy and challenges of carbon nanotube in wastewater and water treatment. Environ Nanotechnol Monit Manag 19:100764. 10.1016/j.enmm.2022.100764

[CR32] Mishra Y, Mishra V, Chattaraj A, Aljabali AAA, El-Tanani M, Farani MR, Huh YS, Serrano-Aroca Ã, Tambuwala MM (2023) Carbon nanotube-wastewater treatment nexus: where are we heading to? Environ Res 238:117088. 10.1016/j.envres.2023.117088 37683781 10.1016/j.envres.2023.117088

[CR33] Mudaliar SB, Bharath Prasad AS (2024) A biomedical perspective of pyocyanin from *Pseudomonas aeruginosa*: its applications and challenges. World J Microbiol Biotechnol 40:90. 10.1007/s11274-024-03889-0 38341389 10.1007/s11274-024-03889-0 PMC10858844

[CR34] Nawar AM, Yahia IS, Al-Kotb MS (2020) Convective self-assembled processed multiwall carbon nanotube thin films for semi-transparent microelectronic applications. J Mater Sci: Mater Electron 31:12127–12136. 10.1007/s10854-020-03759-z

[CR35] Patil S, Nikam M, Patil H, Anokhina T, Kochetkov V, Chaudhari A (2017) Bioactive pigment production by *Pseudomonas* spp. MCC 3145: statistical media optimization, biochemical characterization, fungicidal and DNA intercalation-based cytostatic activity. Process Biochem 58:298–305. 10.1016/j.procbio.2017.05.003

[CR36] Peña-Morán OA, Villarreal ML, Álvarez-Berber L, Meneses-Acosta A, Rodríguez-López V (2016) Cytotoxicity, post-treatment recovery, and selectivity analysis of naturally occurring podophyllotoxins from *Bursera fagaroides* var. *fagaroides* on breast cancer cell lines. Molecules 21. 10.3390/molecules21081013 10.3390/molecules21081013 PMC6274026 27527135

[CR37] Rathinavel S, Priyadharshini K, Panda D (2021) A review on carbon nanotube: an overview of synthesis, properties, functionalization, characterization, and the application. Materials Science and Engineering: B 268

[CR38] Selim MS, Azzam AM, Shenashen MA, Higazy SA, Mostafa BB, El-Safty SA (2024) Comparative study between three carbonaceous nanoblades and nanodarts for antimicrobial applications. Journal of Environmental Sciences 136:594–605. 10.1016/j.jes.2023.02.036 10.1016/j.jes.2023.02.036 37923468

[CR39] Shouman H, Said HS, Kenawy HI, Hassan R (2023) Molecular and biological characterization of pyocyanin from clinical and environmental *Pseudomonas aeruginosa*. Microb Cell Fact 22. 10.1186/s12934-023-02169-0 10.1186/s12934-023-02169-0 PMC10466709 37644606

[CR40] Sonowal L, Gautam S (2024) Advancements and challenges in carbon nanotube-based drug delivery systems. Nano-Structures & Nano-Objects 38:101117. 10.1016/j.nanoso.2024.101117

[CR41] Sun S, Zhou L, Jin K, Jiang H, He Y-W (2016) Quorum sensing systems differentially regulate the production of phenazine-1-carboxylic acid in the rhizobacterium *Pseudomonas aeruginosa* PA1201. Sci Rep 6:30352. 10.1038/srep30352 27456813 10.1038/srep30352 PMC4960564

[CR42] Valentini L, Bon SB, Signetti S, Tripathi M, Iacob E, Pugno NM (2016) Fermentation based carbon nanotube multifunctional bionic composites. Sci Rep 6. 10.1038/srep27031 10.1038/srep27031 PMC4899685 27279425

[CR43] Weise K, Winter L, Fischer E, Kneis D, de la Cruz Barron M, Kunze S, Berendonk TU, Jungmann D, Klümper U (2022) Multiwalled carbon nanotubes promote bacterial conjugative plasmid transfer. Microbiol Spectr 10. 10.1128/spectrum.00410-22 10.1128/spectrum.00410-22 PMC9045119 35384690

[CR44] Zhao J, Wu Y, Alfred AT, Wei P, Yang S (2014) Anticancer effects of pyocyanin on HepG2 human hepatoma cells. Lett Appl Microbiol 58:541–548. 10.1111/lam.12224 24461061 10.1111/lam.12224

[CR45] Zheng S, Li Z, Zhang P, Wang B, Zhang P, Feng Y (2020) Multi-walled carbon nanotubes accelerate interspecies electron transfer between Geobacter cocultures. Bioelectrochemistry 131. 10.1016/j.bioelechem.2019.107346 10.1016/j.bioelechem.2019.107346 31706115

