# Supplementary material for: Multi-walled carbon nanotubes as reusable boosters of pyocyanin production for anticancer research
Source: Appl Microbiol Biotechnol. 2025 Jul 14;109:167. doi: 10.1007/s00253-025-13543-w (PMC12259743; doi:10.1007/s00253-025-13543-w)
Supplement: Supplementary file 1 [file 253_2025_13543_MOESM1_ESM.docx]

**Multi-walled carbon nanotubes as reusable boosters of pyocyanin production for anticancer research**

Joanna Honselmann genannt Humme^1*^, Kamila Dubrowska^1^, Magdalena Perużyńska^2^, Marek Droździk^2^, Radosław Birger^2^, Martyna Jurkiewicz^3^, Tomasz Kędzierski^4^, Ewa Mijowska^4^, Tomasz Idzik^5^, Jacek G. Sośnicki^5^, Elżbieta Filipek^6^, Mateusz Piz^6^, Rafał Rakoczy^1,7^, Adrian Augustyniak^1,7^

^1^ Department of Chemical and Process Engineering, Faculty of Chemical Technology and Engineering, West Pomeranian University of Technology in Szczecin, Szczecin, Poland

^2^ Department of Experimental & Clinical Pharmacology, Pomeranian Medical University in Szczecin, Szczecin, Poland

^3^ Department of Chemical Organic Technology and Polymeric Materials, Faculty of Chemical Technology and Engineering, West Pomeranian University of Technology in Szczecin, Szczecin, Poland

^4^ Department of Nanomaterials Physicochemistry, Faculty of Chemical Technology and Engineering, West Pomeranian University of Technology in Szczecin, Szczecin, Poland

^5^ Department of Organic and Physical Chemistry, Faculty of Chemical Technology and Engineering, West Pomeranian University of Technology in Szczecin, Szczecin, Poland

^6^ Department of Inorganic and Analytical Chemistry, Faculty of Chemical Technology and Engineering, West Pomeranian University of Technology in Szczecin, Szczecin, Poland

^7^ Center for Advanced Materials and Manufacturing Process Engineering (CAMMPE), Szczecin, Poland

Corresponding author: Joanna Honselmann genannt Humme, [joanna_jablonska@zut.edu.pl](mailto:joanna_jablonska@zut.edu.pl)

**Results**

**Figure S1** The influence of different commercial MWCNTs on optical density and viability of *P. aeruginosa* in microplate cultures

**Figure S2** The influence of different commercial MWCNTs on optical density and viability of *P. aeruginosa* in Petri dish cultures (37℃, 48h)

**Figure S3** The characterization of the optimized and control culture: a) biomass to substrate, product to biomass and product to substrate yield and b) pyoverdine production

**Figure S4** A fragment of ^1^H,^1^H NOESY spectrum of a Pyocyanin sample in CD3OD. (The spectrum has been cropped for clarity)

**Figure S5** A fragment of ^1^H,^1^H DQFCOSY spectrum of a Pyocyanin sample in CD_3_OD. (The spectrum has been cropped for clarity)

**Figure S6** A fragment of ^13^C,^1^H COSY spectrum of a Pyocyanin sample in CD_3_OD. (The spectrum has been cropped for clarity)

**Figure. S7** FTIR-ATR analyses of the purified pyocyanin (400-4000 cm^-1^)

**Figure S8** Representative optical microscopy images of normal (L929) and cancer cells after 24 h incubation with indicated concentrations of PYO

**Figure S9** SEM-EDX analysis of the pristine and reused MWCNTs: a) Sigma Aldrich, b) Sigma Aldrich 1^st^ reuse, c) Sigma Aldrich 2^nd^ reuse
